# The Novel MDM4 Inhibitor CEP-1347 Activates the p53 Pathway and Blocks Malignant Meningioma Growth In Vitro and In Vivo

**DOI:** 10.3390/biomedicines11071967

**Published:** 2023-07-12

**Authors:** Yuta Mitobe, Shuhei Suzuki, Yurika Nakagawa-Saito, Keita Togashi, Asuka Sugai, Yukihiko Sonoda, Chifumi Kitanaka, Masashi Okada

**Affiliations:** 1 Department of Molecular Cancer Science, School of Medicine, Yamagata University, 2-2-2 Iida-nishi, Yamagata 990-9585, Japan; 2 Department of Neurosurgery, School of Medicine, Yamagata University, 2-2-2 Iida-nishi, Yamagata 990-9585, Japan; 3 Department of Clinical Oncology, School of Medicine, Yamagata University, 2-2-2 Iida-nishi, Yamagata 990-9585, Japan; 4 Department of Ophthalmology and Visual Sciences, School of Medicine, Yamagata University, 2-2-2 Iida-nishi, Yamagata 990-9585, Japan; 5 Research Institute for Promotion of Medical Sciences, Faculty of Medicine, Yamagata University, 2-2-2 Iida-nishi, Yamagata 990-9585, Japan

**Keywords:** MDMX, HDMX, HDM4, MDM2 antagonist, drug repositioning, drug repurposing, brain tumor

## Abstract

A significant proportion of meningiomas are clinically aggressive, but there is currently no effective chemotherapy for meningiomas. An increasing number of studies have been conducted to develop targeted therapies, yet none have focused on the p53 pathway as a potential target. In this study, we aimed to determine the in vitro and in vivo effects of CEP-1347, a small-molecule inhibitor of MDM4 with known safety in humans. The effects of CEP-1347 and MDM4 knockdown on the p53 pathway in human meningioma cell lines with and without p53 mutation were examined by RT-PCR and Western blot analyses. The growth inhibitory effects of CEP-1347 were examined in vitro and in a mouse xenograft model of meningioma. In vitro, CEP-1347 at clinically relevant concentrations inhibited MDM4 expression, activated the p53 pathway in malignant meningioma cells with wild-type p53, and exhibited preferential growth inhibitory effects on cells expressing wild-type p53, which was mostly mimicked by MDM4 knockdown. CEP-1347 effectively inhibited the growth of malignant meningioma xenografts at a dose that was far lower than the maximum dose that could be safely given to humans. Our findings suggest targeting the p53 pathway with CEP-1347 represents a novel and viable approach to treating aggressive meningiomas.

## 1. Introduction

Meningiomas, which originate from the arachnoid cap cells in the meninges covering the brain and, thus, grow mainly as extra-axial (i.e., outside the brain) tumors [1], are the most common tumor in the central nervous system [2]. While the majority of meningiomas are benign and classified as grade I in the WHO classification system, high-grade tumors (grades II and III) represented by anaplastic and malignant meningiomas account for as high as 20% of all meningiomas [3]. The standard of care for high-grade meningiomas includes surgical resection and radiotherapy; however, despite treatment with these modalities, they frequently recur and/or progress and eventually devastate patients’ lives [4,5,6]. Therefore, pharmacotherapies are needed to manage such aggressive meningiomas.

With recent advances in our understanding of genetic/epigenetic and molecular changes associated with the development and/or progression of meningioma, drugs targeting these changes are attracting increasing attention and are now being exhaustively tested for their anti-meningioma activity in preclinical and clinical studies [7,8,9,10,11,12]. Although some drugs, such as bevacizumab and everolimus, have so far shown promising results, none have yet to exhibit unequivocal clinical efficacy [7,8,9,10,11,12], underscoring the necessity of continued efforts to pursue therapeutic targets from novel perspectives.

*TP53*, the tumor suppressor gene encoding the p53 transcription factor, is the most frequently mutated gene in human cancer. Its mutation results in the loss of the transcriptional activation of p53 target genes involved in, for example, the control of cellular proliferation and apoptosis, which promotes the malignant transformation of cells [13]. Notably, in contrast to the majority of human cancers, a low incidence of p53 mutation has been reported as one of the genetic features of meningioma [14,15], which may have been overlooked in part because of its “negative” nature. In wild-type p53 cancer cells, p53 is functionally inactivated through the deregulation of its negative regulators, such as murine double minute 2 (MDM2) and MDM4, which may inhibit the transcriptional activity of p53 by themselves or promote the MDM2-mediated degradation of p53 by forming a heterodimer [16,17,18]; therefore, the reactivation of the p53 pathway by targeting these negative regulators of p53 represents a rational approach to controlling the growth of meningioma cells with wild-type p53. However, to the best of our knowledge, the effects of “p53-targeting drugs” have not yet been examined in meningioma cells. Therefore, we herein investigated the effects of CEP-1347 on malignant meningioma cells, which we recently characterized as a novel inhibitor of MDM4 protein expression that is capable of activating the p53 pathway in cancer cells expressing wild-type p53 [19,20].

## 2. Materials and Methods

### 2.1. Reagents and Antibodies

CEP-1347 was purchased from TOCRIS Bioscience (Bristol, UK) and dissolved in DMSO to prepare a 0.5 mM stock solution. Staurosporine was from Merck KGaA (Darmstadt, Germany); an antibody (A700-000-T) against MDM4 was from BETHYL (FORTIS LIFE SCIENCES, Waltham, MA, USA); and an antibody against MDM2 (AF1244) was from R&D Systems (Minneapolis, MN, USA). Antibodies against cyclin-dependent kinase inhibitor 1A (CDKN1A, p21^Waf1/Cip1^) (#2947), PUMA (#12450), BAX (#5023), cleaved caspase-3 (#9661), cleaved PARP (#9541), and GAPDH (#5174) were from Cell Signaling Technology, Inc. (Beverly, MA, USA), and an antibody against p53 (sc-126) was from Santa Cruz Biotechnology, Inc. (Santa Cruz, CA, USA).

### 2.2. Cell Culture

IMR90, normal human fetal lung fibroblasts, and IOMM-Lee, a human malignant meningioma cell line expressing wild-type p53, were obtained from the American Type Culture Collection (Manassas, VA, USA) and cultured in Dulbecco’s modified Eagle’s medium supplemented with 10% or 5% fetal bovine serum (FBS), respectively. HKBMM, a human malignant meningioma cell line expressing a mutant p53 (P177L), was obtained from the Riken BioResource Research Center (Tsukuba, Japan) and maintained in Ham’s F12 medium supplemented with 10% FBS. The culture media were supplemented with 100 U/mL penicillin and 100 μg/mL streptomycin. The p53 status of IOMM-Lee and HKBMM cells was examined by cDNA sequencing as described below. IMR90 cells passaged fewer than 8 times were used in experiments. Detailed conditions for the in vitro drug treatment are described in the respective figure legends. In general, the day after cells had been passaged, the required amount of the drug stock solution was added to cells and gently mixed well. This was followed by an incubation at 37 °C in a 5% CO_2_ incubator for the specified time, and cells were then subjected to each assay.

### 2.3. Western Blot Analysis

Western blotting was performed according to a previously reported method [21]. Following the harvesting of cells and washing with ice-cold phosphate-buffered saline (PBS), they were lysed in RIPA buffer (10 mM Tris/HCl (pH 7.4), 0.1% sodium dodecyl sulfate (SDS), 1% Nonidet P-40, 0.1% sodium deoxycholate, 150 mM NaCl, 1 mM EDTA, 10 mM sodium pyrophosphate, 1.5 mM sodium orthovanadate, 10 mM sodium fluoride, and protease inhibitor cocktail set III (FUJIFILM Wako Chemicals, Osaka, Japan)). Lysates were immediately mixed with the same volume of 2 × Laemmli buffer (125 mM Tris/HCl [pH 6.8], 4% SDS, and 10% glycerol) and then boiled at 95 °C for 10 min. A BCA protein assay kit (Thermo Fisher Scientific, Waltham, MA, USA) was used to assess the protein concentrations of the cell lysates. Samples with equal amounts of protein were then resolved by SDS-PAGE and transferred to PVDF membranes. Membranes were probed with the indicated primary antibodies followed by appropriate horseradish peroxidase (HRP)-conjugated secondary antibodies as recommended by the manufacturer of each antibody. Regarding reprobing, the primary antibodies were stripped from the probed membrane using stripping buffer (2% SDS, 100 mM β-mercaptoethanol, and 62.5 mM Tris-HCl (pH 6.8)). Membranes were washed with Tris-buffered saline with Tween 20, blocked with skim milk, and then incubated with the appropriate antibodies. The chemiluminescence of samples was detected using Immobilon Western Chemiluminescent HRP Substrate (Merck KGaA) and the ChemiDoc Touch device (Bio-Rad, Hercules, CA, USA).

### 2.4. Reverse Transcription (RT)-PCR Analyses

RT-PCR was performed according to a previously reported method [22]. Total RNA was extracted from cells using Trizol (Thermo Fisher Scientific). To synthesize first-strand cDNA, total RNA (1 μg) was reverse-transcribed using oligo-dT primers and PrimeScript II RTase (Takara Bio, Inc., Tokyo, Japan). Target genes were amplified with Quick Taq HS DyeMix (Toyobo CO., LTD., Osaka, Japan). The sequences of gene-specific primer sets listed in Table 1 were designed using Primer-BLAST (https://www.ncbi.nlm.nih.gov/tools/primer-blast/, accessed on 10 May 2019).

### 2.5. cDNA Sequencing of p53

cDNA sequencing was performed to evaluate the p53 status of IOMM-Lee and HKBMM cells. Two microliters of cDNA was amplified by RT-PCR using KOD-plus-Neo DNA polymerase (TOYOBO), with 15 pmol of each primer: 5′-TGG ATT GGC AGC CAG ACT GC-3′ (sense) and 5′-TGA CGC ACA CCT ATT GCA AGC-3′ (antisense). PCR conditions were as follows: 94 °C for 2 min, followed by 34 cycles at 98 °C for 10 s and 68 °C for 40 s. p53-specific primer sequences were as follows: 5′-AAT CAA CCC ACA GCT GCA CAG-3′ (antisense) and 5′- CAG GCA CAA ACA CGC ACC TC-3′ (antisense), and the two primers described above were extracted from the database (https://www.ncbi.nlm.nih.gov/nucleotide/NM_000546.6?report=genbank&log$=nuclalign&blast_rank=1&RID=4BUCMK6D013, accessed on 7 February 2023) (Hokkaido System Science Co., Ltd., Hokkaido, Japan). After PCR amplification, PCR products were purified using the FastGene Genomic DNA Extraction Kit (NIPPON Genetics Co., Ltd., Tokyo, Japan), and sequenced by the ABI 3500 Genetic Analyzer (Thermo Fisher Scientific). Sequences were compared with the wild-type p53 sequence, which was extracted from the p53 GenBank database (www.ncbi.nlm.nih.gov, accessed on 17 January 2023; accession no. NM_000546).

### 2.6. Gene Silencing by siRNA

siRNAs against human MDM4 (#1: HSS106417, #2: HSS106418, and #3: HSS106419), human p53 (#1: HSS186390 and #2: HSS186391), and Stealth RNAi siRNA Negative Control Med GC Duplex #2 were from Thermo Fisher Scientific. Cells were transiently transfected with one of the siRNAs against MDM4 (siMDM4; 120–160 pmol per 6 cm dish) or p53 (sip53; 120–160 pmol per 6 cm dish) or with the control siRNA (siCt; 120–160 pmol per 6 cm dish) using Lipofectamine RNAiMAX (Thermo Fisher Scientific) according to the manufacturer’s instructions.

### 2.7. Trypan Blue Dye Exclusion Assay

Trypan Blue solution (T8154, Merck KGaA) was used to assess the numbers of viable and dead cells [20]. Adherent and non-adherent cells in culture dishes were collected, centrifuged, resuspended in PBS, and stained with Trypan blue solution (final, 0.2%) for 1 min. A hemocytometer identified viable and dead cells based on their ability and inability, respectively, to exclude trypan blue.

### 2.8. Propidium Iodide (PI) Incorporation Assay

The PI incorporation assay was used to assess cell death [22]. Cells were incubated in situ with PI (final concentration, 1 μg/mL) and Hoechst33342 (final concentration,10 μg/mL) at 37 °C for 5 min in the CO_2_ incubator. To evaluate the percentage of PI-positive (dead) cells among Hoechst-positive (total) cells, fluorescent images were obtained using a fluorescence microscope (CKX41; Olympus, Tokyo, Japan) equipped with an iPhone 7 and scored. More than 170 cells were counted to assess the percentage of PI-positive cells.

### 2.9. Hoechst33342 Staining to Detect Nuclear Condensation

Hoechst33342 staining was used to visualize nuclear condensation. Cells plated on coverslips were fixed with 4% paraformaldehyde (*w*/*v*) in PBS at room temperature for 10 min. Fixed cells were then incubated with Hoechst33342 (500 ng/mL in PBS) at room temperature for 20 min. Coverslips were mounted using DABCO solution (0.5% DABCO (D27802, Merck KGaA), 90% glycerol in 10 mM Tris/HCl (pH 7.4)). Fluorescent images were obtained using a BZ-X700 fluorescence microscope (KEYENCE CORPORATION, Osaka, Japan).

### 2.10. Colony Formation Assay

To assess clonogenic outgrowth, we performed a colony formation assay according to a previously reported method [23]. In brief, cells were seeded at a low, colony-forming density (200 cells/12-well plate for IOMM-Lee and 300 cells/12-well plate for HKBMM). After being cultured as described in each figure legend, cells were fixed with paraformaldehyde (4% *w*/*v*), followed by staining with crystal violet (0.1% *w*/*v*). Colonies comprising 50 or more cells derived from a single cell were counted using a microscope.

### 2.11. Mouse Study

Mouse xenograft studies were conducted as previously described [24,25]. After assessing cell viability using the dye exclusion method, 1×10^6^ viable IOMM-Lee cells suspended in 200 µL PBS were subcutaneously implanted bilaterally into the flanks of 5- to 7-week-old male BALB/cAJcl-nu/nu mice (CLEA Japan, Tokyo, Japan) anesthetized by an intraperitoneal injection of butorphanol, midazolam, and medetomidine (5, 4, and 0.3 mg per kg body weight, respectively). Tumor volumes were assessed by measuring tumor diameters in two perpendicular axes of tumors with a caliper and calculated as 1/2 × (larger diameter) × (smaller diameter)^2^.

Regarding the systemic administration of CEP-1347, the stock solution of CEP-1347 (0.75 mg/mL in DMSO) was diluted with PBS to prepare a 200 μL solution for each injection. CEP-1347 solutions were intraperitoneally injected into nude mice. All vehicle- and CEP-1347-treated mice received an equal volume of DMSO per body weight (2.0 mL/kg). All animal experiments were performed under a protocol approved by the Animal Research Committee of Yamagata University (R4038).

### 2.12. Statistical Analysis

Results are shown as means ± standard deviation (SD). Data were analyzed using Student’s *t*-test for comparisons between two groups. *p* < 0.05 indicated a significant difference and was shown with asterisks in the figures.

## 3. Results

### 3.1. CEP-1347 Preferentially Inhibits the Growth of Malignant Meningioma Cells Expressing Wild-Type p53 over Those Expressing a Mutant p53 Protein

**Figure 1:**
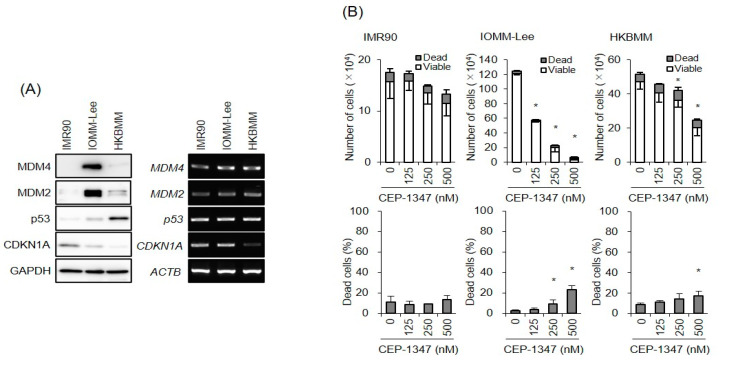
CEP-1347 preferentially inhibits the growth of malignant meningioma cells expressing wild-type p53 over those expressing a mutant p53 protein. (**A**) The status of the p53 pathway in IMR90 normal human fibroblasts and malignant meningioma cells with wild-type (IOMM-Lee) and mutated (HKBMM) p53. The indicated cells maintained under their respective culture conditions were subjected to Western blot (left panels) and RT-PCR (right panels) analyses for the expression of murine double minute (MDM) 4, MDM2, p53, and cyclin dependent kinase inhibitor 1A (CDKN1A, p21^Waf1/Cip1^). (**B**) IMR90 and malignant meningioma cells (IOMM-Lee and HKBMM) treated for 3 days with CEP-1347 at the indicated concentrations were subjected to the trypan blue dye exclusion assay to measure the numbers of viable and dead cells (upper panels) as well as the percentage of dead cells (lower panels). Values represent the means ± standard deviations (SDs) of triplicate samples of a representative experiment. * *p* < 0.05 vs. cells treated without CEP-1347 (i.e., at 0 nM) by Student’s *t*-test. Similar results were obtained from more than two independent biological replicates.

We previously demonstrated that CEP-1347, as an inhibitor of MDM4, more efficiently suppressed the growth of glioma cells expressing wild-type p53 than those expressing a mutant p53 [20]. To investigate whether the same is true with meningioma cells, we examined the effects of CEP-1347 on the growth of two malignant meningioma cell lines, IOMM-Lee and HKBMM, which express wild-type and a mutant (P177L) p53, respectively. While MDM2 and MDM4 mRNA levels were similar in these two meningioma cell lines and IMR90 normal human fibroblasts, MDM2 and MDM4 protein levels were higher in IOMM-Lee cells than in the other two cell lines (Figure 1A), suggesting that some post-transcriptional mechanism of upregulating the negative regulators of p53 was operative to inactivate the p53 pathway in IOMM-Lee cells. When the two meningioma cell lines were treated with CEP-1347 at concentrations up to 500 nM, a range of concentrations that do not significantly inhibit the growth of normal human fibroblasts (IMR90), IOMM-Lee cells were more sensitive to growth inhibition by CEP-1347 than HKBMM cells (Figure 1B), which supports the p53 status being predictive of the sensitivity of cells to CEP-1347.

**Figure 2:**
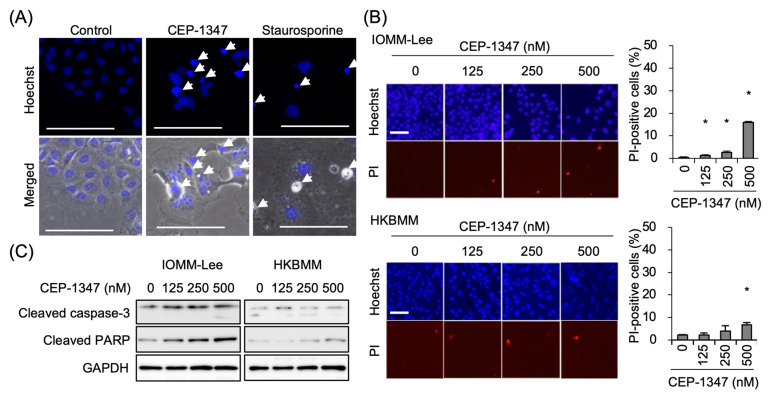
Induction of apoptotic cell death and caspase activation by CEP-1347. (**A**) Representative images of Hoechst33342-stained IOMM-Lee nuclei following a treatment without (left), with 500 nM CEP-1347 (middle), or with 10 nM staurosporine (right) for 2 days. Condensed nuclei (arrows) are highlighted. Bars: 100 μm. (**B**) Cells treated with the indicated concentrations of CEP-1347 for 3 days were subjected to the propidium iodide (PI) incorporation assay. Representative fluorescence images of Hoechst- (upper rows) and PI- (lower rows) positive cells are shown. Bars: 100 μm. Values represent means + SDs of triplicate samples of a representative experiment. * *p* < 0.05 vs. cells treated without CEP-1347 (i.e., at 0 nM) by Student’s *t*-test. (**C**) Activation of the caspase pathway. Cells treated as in (**B**) were subjected to Western blot analysis for the expression of the indicated proteins. Similar results were obtained from more than two independent biological replicates.

Since the inhibition of IOMM-Lee cell growth by CEP-1347 was accompanied by modest increase in dead cells (Figure 1B), we characterized cell death induced by CEP-1347 using different approaches. The results obtained showed that cells undergoing cell death after the CEP-1347 treatment had condensed nuclei suggestive of apoptosis (Figure 2A, middle), which was similar to the morphology of cell death after the treatment with the prototypical apoptosis inducer, staurosporine [26] (Figure 2A, right), and eventually lost the ability to exclude the vital dye PI (Figure 2B). We also found that the increase in dead cells was accompanied by up-regulated expression of cleaved caspase-3 and cleaved PARP (Figure 2C).

**Figure 3:**
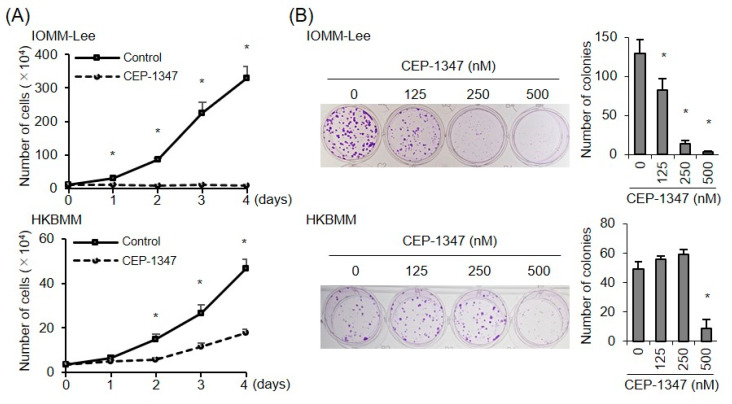
Suppression of the clonogenic outgrowth of malignant meningioma cells by CEP-1347. (**A**) Cell-growth curves were assessed by the trypan blue dye exclusion assay for the indicated cell lines treated without (Control) or with 500 nM CEP-1347. (**B**) IOMM-Lee and HKBMM cells treated with the indicated concentrations of CEP-1347 for 3 days were cultured for another 6 days in the absence of CEP-1347 for the colony formation assay. Representative images (left panels) and the number of colonies (right graphs) are shown. Values represent the means + SDs of triplicate samples of a representative experiment. * *p* < 0.05 vs. cells treated without CEP-1347 (i.e., at 0 nM) by Student’s *t*-test. Similar results were obtained from more than two independent biological replicates.

These results indicated that apoptotic cell death contributed, to some extent, to the CEP-1347-induced inhibition of malignant meningioma cell growth. We then investigated whether CEP-1347 inhibited the growth of malignant meningioma cells in a potent and sustained manner that was clinically significant. The time-course analysis of cell growth after the CEP-1347 treatment indicated that CEP-1347 almost completely inhibited the growth of IOMM-Lee cells, whereas it only modestly suppressed that of HKBMM cells (Figure 3A). The results of the colony formation assay revealed that CEP-1347 significantly inhibited the clonogenic survival of IOMM-Lee cells at a concentration as low as 125 nM but that of HKBMM cells only at 500 nM (Figure 3B). Collectively, these results suggested that CEP-1347 exerted growth inhibitory effects on malignant meningioma cells that were dependent on the p53 status of cells.

### 3.2. CEP-1347 Reduces the Expression of MDM4 and Activates the p53 Pathway in Malignant Meningioma Cells with Wild-Type p53

**Figure 4:**
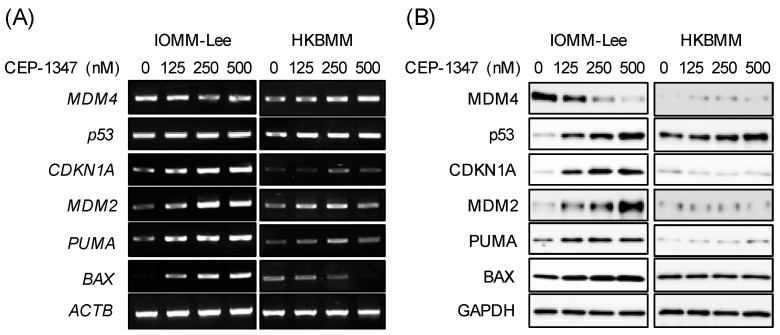
CEP-1347 reduces the expression of MDM4 and activates the p53 pathway in malignant meningioma cells with wild-type p53. IOMM-Lee and HKBMM cells treated with the indicated concentrations of CEP-1347 for 2 days were subjected to RT-PCR (**A**) and Western blot (**B**) analyses for the expression of MDM4, p53, CDKN1A, MDM2, PUMA, and BAX. Similar results were obtained from more than three independent biological replicates.

**Figure 5:**
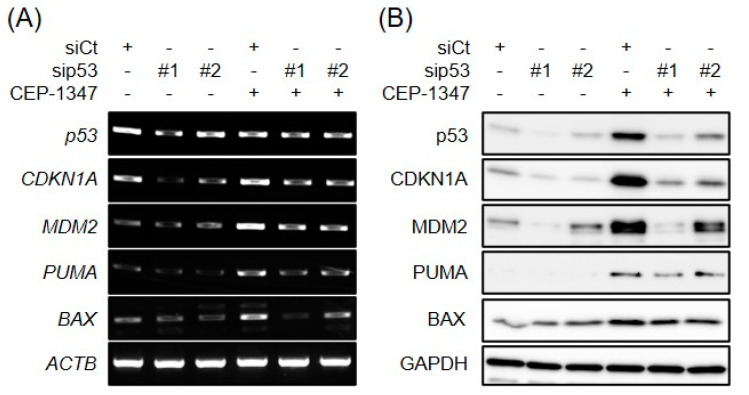
CEP-1347 activates the p53 pathway in a p53-dependent manner. IOMM-Lee cells were transiently transfected with the indicated siRNA against p53 (#1 and #2) or with control siRNA (siCt). After being cultured for 1 day, cells were treated without or with 500 nM CEP-1347 for 1 and 2 additional days and then subjected to RT-PCR (**A**) and Western blot (**B**) analyses, respectively. Similar results were obtained from more than two independent biological replicates.

Based on the different sensitivities of malignant meningioma cell lines to CEP-1347, we investigated whether these differential effects may be attributed to the ability and inability of CEP-1347 to activate the p53 pathway in these cell lines. The treatment of IOMM-Lee cells with CEP-1347 resulted in a concentration-dependent decrease in MDM4 protein but not mRNA expression levels and also a reciprocal increase in p53 protein expression levels (Figure 4A,B), which is consistent with our previous findings on cell lines established from retinoblastoma and glioblastoma [19,20], cancer types associated with low incidence of p53 mutation [27,28]. In contrast and as expected, CEP-1347 failed to affect the expression level of MDM4 or p53 in HKBMM cells (Figure 4A,B). Importantly, the up-regulated expression of p53 in IOMM-Lee cells after the CEP-1347 treatment was accompanied by increases in mRNA and protein expression levels of p53 target genes such as *CDKN1A/p21* and *MDM2* (Figure 4A,B). Consistent with the earlier result showing that CEP-1347 induced apoptosis in IOMM-Lee cells, the mRNA and protein expression levels of *PUMA* and *BAX*, pro-apoptotic target genes of p53, also increased in parallel with p53 (Figure 4A,B). To investigate whether CEP-1347 induced the expression of these p53 target genes via p53, we examined the dependence of their expression on p53. In p53 knockdown cells (Figure 5A,B), CEP-1347 failed to efficiently increase the mRNA or protein expression levels of CDKN1A/p21, MDM2, PUMA, or BAX, suggesting that the CEP-1347 activation of p53 mediated the increased expression of these genes.

### 3.3. Reductions in MDM4 Expression Levels Activate the p53 Pathway in Malignant Meningioma Cells with Wild-Type p53

**Figure 6:**
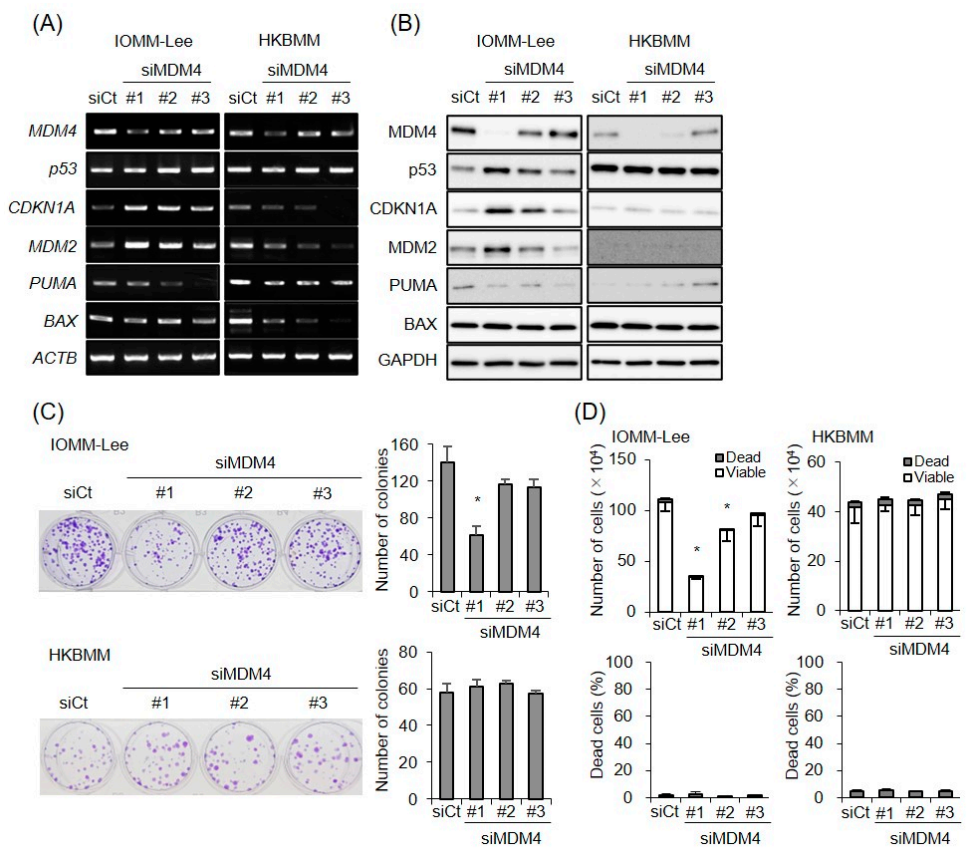
Reduction in MDM4 expression leads to p53 activation in malignant meningioma cells with wild-type p53. (**A**,**B**) IOMM-Lee and HKBMM cells were transiently transfected with the indicated siRNA against MDM4 (#1, #2, and #3) or with control siRNA (siCt). After being cultured for 2 and 3 days, cells were subjected to RT-PCR (**A**) and Western blot (**B**) analyses, respectively. (**C**) Cells transiently transfected as in (**A**) were cultured for 8 days for the colony formation assay. Representative images (left panels) and the number of colonies (right graphs) are shown. (**D**) Cells were transiently transfected as in (**A**). After 3 days, the transfected cells were subjected to the trypan blue dye exclusion assay to assess the numbers of viable and dead cells (upper graphs) as well as the percentage of dead cells (lower graphs). Values represent the means ± SDs of triplicate samples of a representative experiment. * *p* < 0.05 vs. siCt-transfected cells by Student’s *t*-test. Similar results were obtained from more than two independent biological replicates.

We investigated whether a CEP-1347-induced reduction in MDM4 expression levels mediated the activation of the p53 pathway in meningioma cells. In contrast to MDM2, which directly regulates the expression of p53 through a physical interaction, MDM4 exerts the same effect indirectly in an MDM2-dependent manner [17], rendering its role in p53 regulation more likely to be dependent on the cell type. Since the role of MDM4 in meningioma cell biology remains unknown, we investigated whether its expression was actually required to prevent the activation of the p53 pathway in meningioma cells. The introduction of siRNAs targeting different sites on MDM4 mRNA reduced MDM4 expression to varying degrees, and, depending on the efficiency of the MDM4 knockdown, p53 protein levels increased in IOMM-Lee cells (Figure 6A,B), demonstrating that the endogenous expression of MDM4 was required to keep p53 inactive in these cells. The clonogenic growth of IOMM-Lee cells was consistently inhibited when MDM4 expression was efficiently knocked down (Figure 6C), corroborating the idea that MDM4 contributes to the unrestricted growth of meningioma cells with wild-type p53 by preventing the activation of the p53 pathway. Therefore, the present results suggested that CEP-1347 inhibited the growth of IOMM-Lee cells by activating p53 through a reduction in the expression of MDM4. However, we found that the MDM4 knockdown-mediated activation of p53 up-regulated the expression of CDKN1A/p21 and MDM2 but not that of PUMA or BAX (Figure 6A,B). Consistent with this result, the knockdown of MDM4, in contrast to CEP-1347, failed to induce cell death in IOMM-Lee cells (Figure 6D). These results suggested that CEP-1347 not only reduced the expression of MDM4 but also elicited a signal that enhanced the transcriptional activity of p53 towards its pro-apoptotic target genes.

### 3.4. Anti-Tumor Activity of CEP-1347 against Malignant Meningioma In Vivo

**Figure 7:**
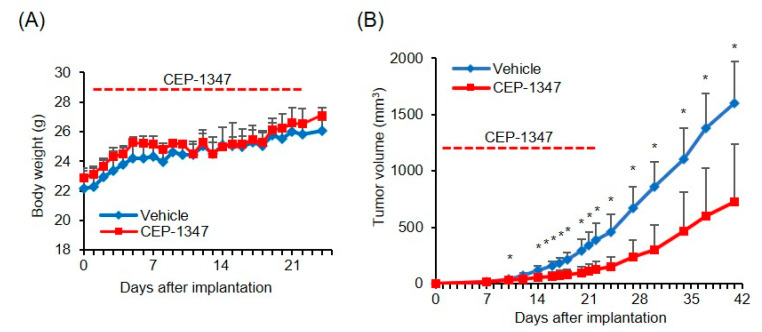
Anti-tumor activity of CEP-1347 against malignant meningioma in vivo. After randomization according to body weight, mice (four for each group) implanted subcutaneously with 1 × 10^6^ viable IOMM-Lee cells were treated with a daily intraperitoneal injection of control vehicle or CEP-1347 (1.5 mg/kg) for 21 days starting on the next day of implantation. The body weights of mice (**A**) and tumor volumes (**B**) were measured at the indicated time points. Values represent the means + SDs of each treatment group. * *p* < 0.05 vs. mice treated with vehicle by Student’s *t*-test.

We assessed the anti-tumor activity of CEP-1347 in vivo using a xenograft model of malignant meningioma. Mice implanted with IOMM-Lee cells were treated daily with the intraperitoneal administration of CEP-1347, which was initiated on the day after implantation and was continued safely for 3 weeks without any evidence of body weight fluctuations (Figure 7A). Subcutaneous tumors grew at a similar pace in CEP-1347- and vehicle-treated mice for approximately the first 10 days. However, the growth of tumors in CEP-1347-treated mice gradually slowed thereafter, and, after 2 weeks of treatment and longer, the volume of CEP-1347-treated tumors was significantly smaller than that of vehicle-treated tumors (Figure 7B). These results indicated that CEP-1347 inhibited the growth of malignant meningioma cells with wild-type p53 not only in vitro but also in vivo.

## 4. Discussion

The management of aggressive meningiomas that resist standard treatment with surgery and radiotherapy, as exemplified by malignant meningiomas, is in desperate need of novel treatment strategies. A number of agents are currently being developed for this purpose, but all are still in the experimental stages, which warrants further approaches that target the hitherto unknown vulnerabilities of aggressive meningiomas [7,8,9,10,11,12]. We herein investigated the potential of the CEP-1347-mediated activation of wild-type p53 as a novel therapeutic approach in the management of malignant meningiomas.

CEP-1347 was originally identified as an inhibitor of mixed lineage kinases that suppresses the JNK pathway and thereby promotes neuronal survival by preventing apoptosis [29,30]. The subsequent demonstration of its ability to prevent neuronal apoptosis not only in vitro but also in vivo eventually led to a phase 3 clinical trial to test its efficacy in the treatment of early Parkinson’s disease [31,32,33]. Although CEP-1347 failed to show efficacy in the clinical trial, the findings obtained highlighted the safety of the long-term oral administration of CEP-1347 in humans [33]. On the other hand, limited information is currently available on CEP-1347 as a cancer chemotherapeutic. However, we herein demonstrated for the first time that, through its ability to inhibit JNK, which is required for the maintenance of cancer stem cells, CEP-1347 is capable of targeting cancer stem cells both in vitro and in vivo [24]. Our subsequent examination of its effects particularly on non-stem cancer cells, unexpectedly revealed that MDM4-expressing retinoblastoma cells with wild-type p53 were selectively sensitive to the growth inhibitory effects of CEP-1347 and also that CEP-1347 down-regulated MDM4 protein expression and activated the p53 pathway to suppress the growth of these cells [19]. We also demonstrated, using cell lines derived from glioblastoma, that CEP-1347 inhibited MDM4 in glioblastoma cells and that the down-regulation of MDM4 was sufficient to activate the p53 pathway, suggesting that CEP-1347 activated the p53-dependent growth inhibitory signal through the suppression of MDM4 [20]. To identify a novel pharmacotherapy for aggressive meningiomas with the intention to further characterize CEP-1347 as an MDM4 inhibitor, we herein examined its effects on malignant meningioma cells, because meningiomas reportedly have a low incidence of p53 mutation and, thus, may have functionally inactivated p53, similar to retinoblastoma and glioblastoma [14,15]. The results obtained indicated that CEP-1347 activated the p53 pathway as an MDM4 inhibitor and suppressed the growth of malignant meningioma cells expressing wild-type p53 and MDM4. One unexpected and important result of the present study is that even though the CEP-1347 treatment and MDM4 knockdown were similar in that they both increased the expression of p53, they activated the pathways downstream of p53 in a differential manner; the CEP-1347 treatment induced both apoptosis and the expression of pro-apoptotic p53 target genes, such as PUMA and BAX, whereas the knockdown of MDM4 did not. Target gene selection by p53 is known to be controlled by a multi-faceted regulatory network involving, for example, the post-translational modification of p53, cofactors, and chromatin remodeling proteins [34]. Therefore, CEP-1347 may contribute to the activation of p53 by acting on one or more of the components of the network regulating p53 target gene selection besides reducing MDM4 expression. Since the serine 46 phosphorylation and lysine 120 acetylation of p53 have been reported to favor the activation of pro-apoptotic target genes, such as PUMA and BAX [35], it would be intriguing to surmise that CEP-1347 modulates these post-translational modifications of p53. Further in this regard, it might also be interesting to investigate, for instance, whether a combination with HDAC inhibitors that reportedly augment the lysine 120 acetylation of p53 enhances the apoptosis-inducing activity of CEP-1347 [36]. Another important result of this study is the inhibitory activity of CEP-1347 on “bulk tumor growth” in vivo, which is predominantly driven by the proliferation of non-stem cancer cells. The growth of xenograft tumors of malignant meningioma cells was effectively inhibited by the systemic administration of CEP-1347 even at a dose that was approximately 1/10 of the mouse equivalent of the dose of CEP-1347 administered safely to humans for 2 years in the aforementioned clinical trial for Parkinson’s disease [24,33]. Therefore, the present results provide encouraging preclinical evidence for the potential of CEP-1347 as a candidate therapeutic for aggressive meningiomas. In our previous study, we failed to detect any discernible effects of the systemic administration of CEP-1347 on the bulk tumor growth of glioblastoma xenografts while, at the same time, it effectively eliminated glioma stem cells residing in xenograft tumors [24]. However, this apparent lack of an effect is most likely because the period of drug administration and observation (10 days) was not long enough, given that the growth inhibitory effects of CEP-1347 only became discernible after 10 days of the administration of CEP-1347 in the present study (Figure 7B). It is important to note that, while this study was in progress, the anti-tumor activities of MLK3 inhibitors including CEP-1347 were demonstrated in a preclinical mouse xenograft model of triple-negative breast cancer [37]. Since the serum-cultured, triple-negative breast cancer cell line used in that study (MDA-MB 468) has been shown to have a mutation in p53 [38], the results imply that CEP-1347 could possibly exert its growth inhibitory effects on non-stem cancer cells in a p53-independent manner at least in some cancer types such as breast cancer. However, the marked difference in sensitivity to CEP-1347 between IOMM-Lee and HKBMM cells in the present study suggests that the p53-independent mechanism does not play a major role in the CEP-1347 inhibition of meningioma growth.

Extensive efforts have been made to therapeutically exploit molecular aberrations associated with aggressive meningiomas; however, no studies have focused on p53, which is only infrequently mutated in meningiomas. To the best of our knowledge, this is the first study to show the potential of wild-type p53 as an actionable therapeutic target to treat aggressive meningiomas. p53 is considered to be inactivated either genetically or functionally in virtually all human cancers [16,39]. The mechanisms underlying the functional inactivation of p53 in meningiomas currently remain unknown. However, it would be intriguing to note that merlin, the product of the *NF2* gene, was shown to contribute to the stabilization of p53 through the degradation of MDM2 [40]. Another study found that the re-introduction of merlin into merlin-deficient schwannoma cells increased p53 levels and its activity and that the treatment of merlin-deficient schwannoma cells with an MDM2 antagonist (nutlin-3) reduced cell survival in vitro and tumor growth in vivo [41]. Since alterations in the *NF2* gene are the most common genetic abnormality in meningiomas and occur more frequently in aggressive meningiomas [42,43,44], these findings suggest that the loss of merlin/NF2 promotes the development and progression of meningiomas through the functional inactivation of the p53 pathway, which provides support for the therapeutic targeting of wild-type p53 in aggressive meningiomas. In this regard, we examined the effects of RG7112, an orally available, small molecule MDM2 antagonist that has been clinically developed [45,46], on the cells used in the present study. We found that the IC_50_s of RG7112 for malignant meningioma cells (IOMM-Lee) and normal fibroblasts (IMR90) were higher and lower, respectively, than those of CEP-1347 [47], which suggested that CEP-1347 may be superior to MDM2 antagonists as a therapeutic activator of p53 at least for meningiomas expressing MDM4, the presumed target of CEP-1347. There is currently no information on the percentage of meningiomas that express MDM4. Since the present results and previous findings indicated that the MDM4 protein could be overexpressed, due most likely to post-transcriptional regulation, even in the absence of gene amplification and/or transcript overexpression (Figure 1A) [20], an immunohistochemical biomarker analysis of MDM4 expression in surgical specimens of meningiomas may facilitate the selection of patients more likely to benefit from the CEP-1347 treatment in its future clinical application.

In summary, we have herein demonstrated that CEP-1347, as an inhibitor of MDM4 protein expression, induced the expression of p53 target genes involved in the negative regulation of cellular proliferation and survival in malignant meningioma cells with wild-type p53, thereby inhibiting their growth in vitro. We also showed in a mouse xenograft model of meningioma that the anti-tumor activity of CEP-1347 was evident even at a very low dose within its clinically relevant dose range. Therefore, the present study provides the first evidence for wild-type p53 constituting an attractive therapeutic target for aggressive meningiomas and CEP-1347 representing a valid choice for the purpose of targeting p53 in these tumors.

## Figures and Tables

**Table 1:** PCR primer sequences.

Gene Name	Forward	Reverse
*MDM4*	AGGTACGACCAAAACTGCCG	CTGCACTTTGCTTCAGTTGGT
*MDM2*	GGTGCTGTAACCACCTCACA	TGAGTCCGATGATTCCTGCTG
*TP53*	ACAACGTTCTGTCCCCCTTG	CTCCGTCATGTGCTGTGACT
*CDKN1A*	GGGATTTCTTCTGTTCAGGCG	TGGTAGAAATCTGTCATGCTGGT
*PUMA*	TACGAGCGGCGGAGACAAG	AGCACAACAGCCTTTCCTGA
*BAX*	CGGGGAGCAGCCCAGA	GGCAGCCCCCAACCAC
*ACTB*	CCCATGCCATCCTGCGTCTG	CGTCATACTCCTGCTTGCTG

## Data Availability

Data are contained within the article.
